# The Controversial Role of Irisin in Clinical Management of Coronary Heart Disease

**DOI:** 10.3389/fendo.2021.678309

**Published:** 2021-07-01

**Authors:** Wen-Lu Ou-Yang, Bei Guo, Feng Xu, Xiao Lin, Fu-Xing-Zi Li, Su-Kang Shan, Feng Wu, Yi Wang, Ming-Hui Zheng, Qiu-Shuang Xu, Ling-Qing Yuan

**Affiliations:** ^1^ Department of Metabolism and Endocrinology, National Clinical Research Center for Metabolic Diseases, Hunan Provincial Key Laboratory of Metabolic Bone Diseases, The Second Xiangya Hospital, Central South University, Changsha, China; ^2^ Department of Radiology, The Second Xiangya Hospital, Central South University, Changsha, China; ^3^ Department of Pathology, The Second Xiangya Hospital, Central South University, Changsha, China

**Keywords:** irisin, myokine, coronary heart disease, infarction, atherosclerosis

## Abstract

Irisin, a PGC1α-dependent myokine, was once believed to have beneficial effects induced by exercise. Since its first discovery of adipose browning in 2012, multiple studies have been trying to explore the metabolic functions of irisin, such as glucose and lipid metabolism. However, recently many studies with irisin concentration measuring were doubt for methodological problems, which may account for the continuous inconsistencies. New tools like recombinant irisin and gene-knockout mice are required to reconfirm the questioned functions of irisin. In this paper, we make a critical introduction to the latest researches concerning the relationship between irisin and coronary heart disease, which includes atherosclerosis, stable angina pectoris and acute coronary syndromes. These studies provided various controversial evidence of short and long-term monitoring and therapeutic effect from molecular cellular mechanisms, *in vivo* experiments and epidemiological investigation. But with ambiguities, irisin still has a long way to go to identify its functions in the clinical management.

## Introduction

### Irisin

Irisin is a relatively newly discovered myokine, a small protein with 112 amino acids. Its precursor, fibronectin type III domain-containing protein 5 (FNDC5), is regulated by the transcription factor PGC1α (1). In human, irisin is mainly secreted by cardiomyocytes and skeletal muscle cells, and a small amount is also distributed in adipose tissue, brain, liver, spleen, testis and other tissue (2). Irisin is highly conserved among different species, homology of which between mouse and human irisin is almost 100% (1). However, Raschke et al. found that start codon of FNDC5 gene is different, and doubted whether it’s appropriate to translate all the beneficial effect observed in mice to humans (3).

The production level of irisin is affected by many factors. Exercise, cold, age, BMI, glucose, lipid metabolism level, and other cytokines are all related to its serum concentration (4–8). It was initially widely considered as an exercise-induced myokine, whose circulating level was affected by type and frequency of physical activity (1, 9, 10). Jedrychowski’s study (11) validated it by quantitation mass spectrometry (MS). However, inconsistencies emerged to doubt whether the stimulation of exercise worked. Browning of white adipose tissue (WAT), a major effect of irisin, was observed only in inguinal WAT of training mice and attenuated in FNDC5 knockout mice (12, 13), while cold exposure was effective for all adipose depots in murine (14). When it comes to human, current evidence are not qualified enough to confirm the irisin’s responses to exercise (15–20). The disagreement may blame to the insufficient detection accuracy considering the minor amounts of irisin’s expression (0.3 ng/mL in mice (21) and 3-4 ng/mL in humans (11) of serum level measured by MS) (15, 22–24).

**Figure 1:**
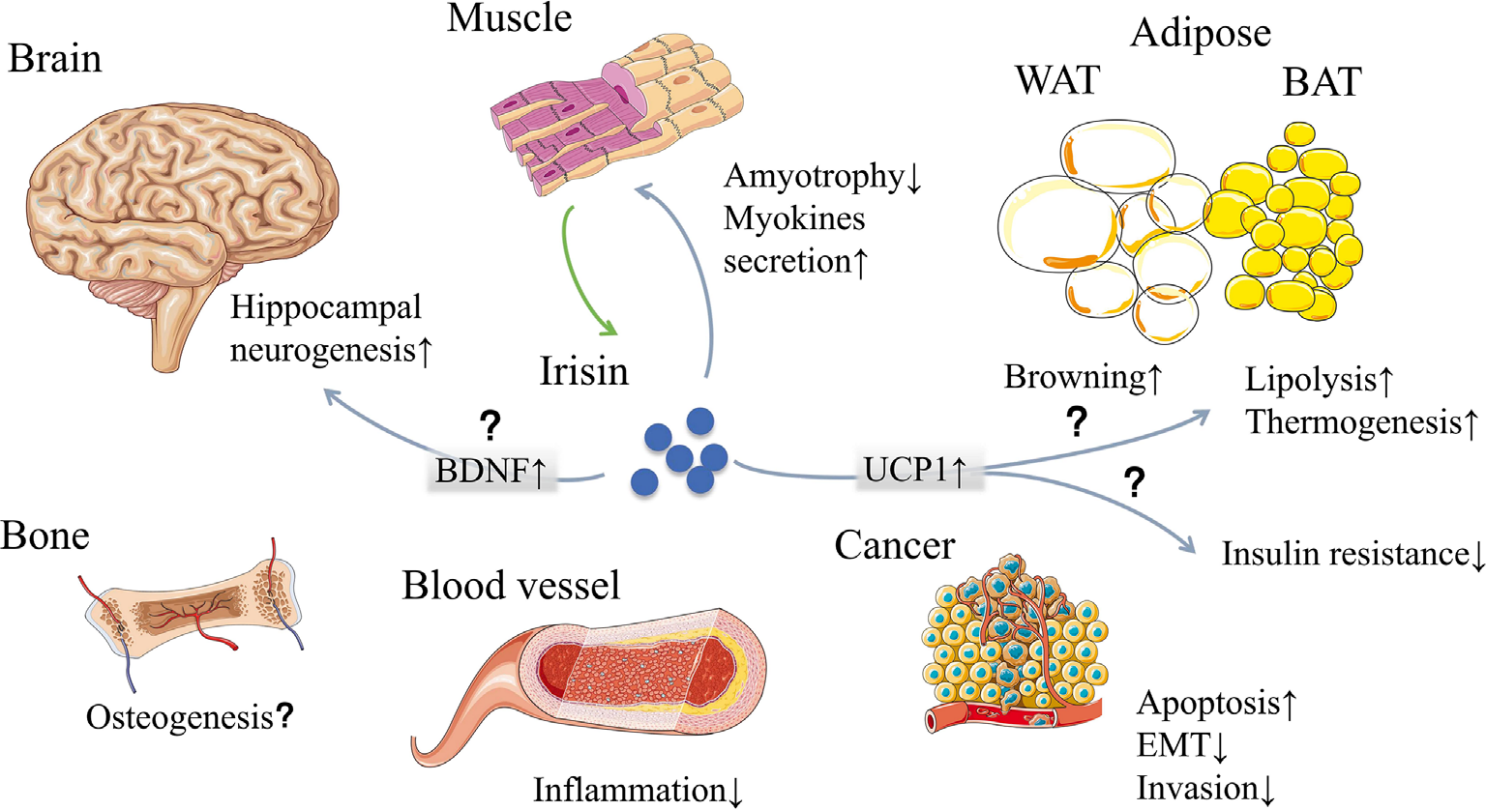
Irisin, mainly secreted by muscle, may have effects on multiple organs and tissues. The classic functions of irisin are browning white adipose tissue and generating heat. Other effects include muscle feedback, neurogenesis, osteogenesis, inflammation reduction, cancer suppression and so on. However, some of the functions are now facing challenges due to the measuring problems and interspecies genetic differences. WAT, white adipose tissue; BAT, brown adipose tissue; BDNF, brain-derived neurotrophic factor; UCP1, uncoupling protein 1; EMT, epithelial-mesenchymal transition. [The elements were produced using Servier Medical Art (https://smart.servier.com/) with some adaption].

**Figure 2:**
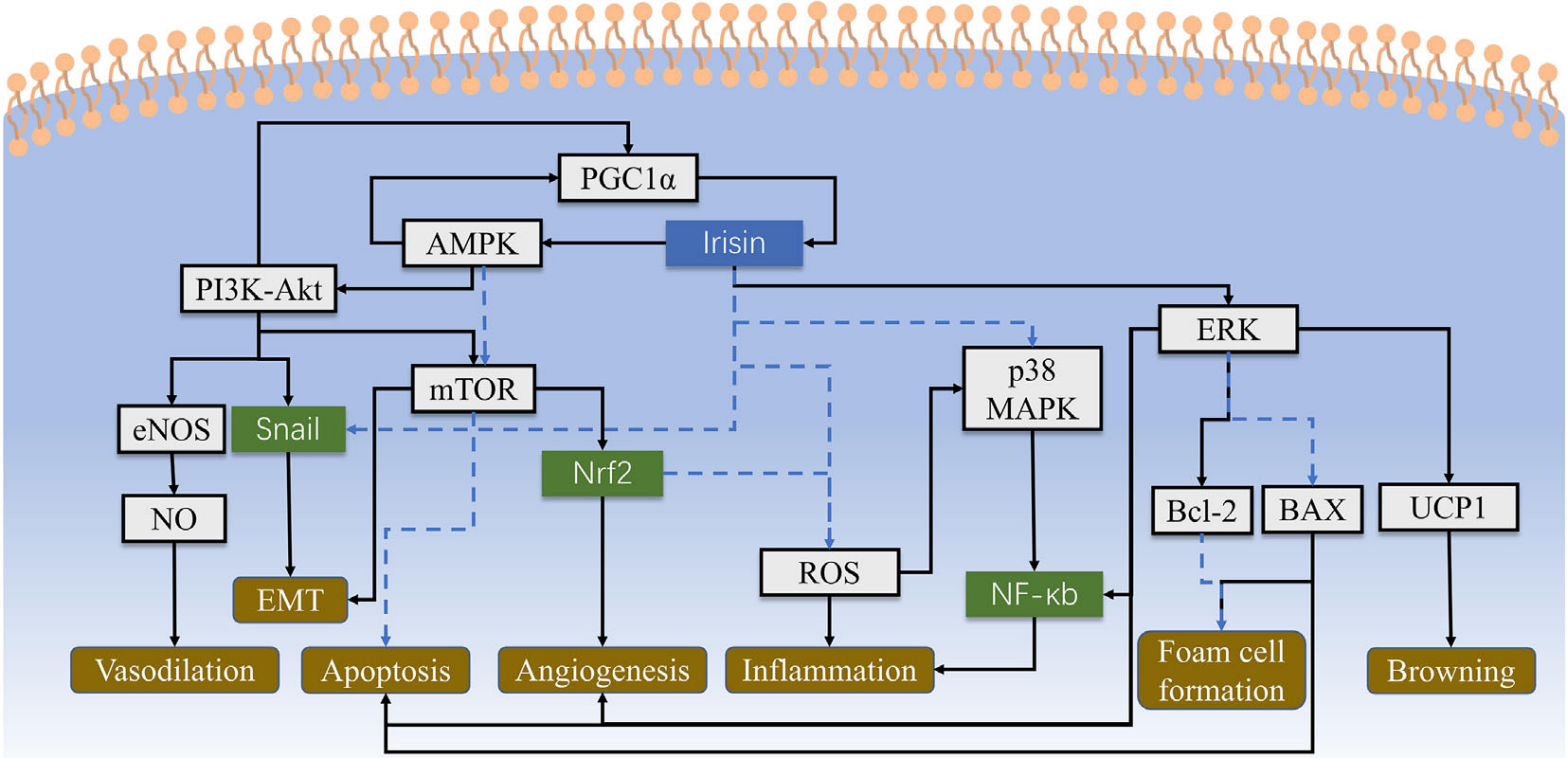
The cell signaling pathways of irisin are intricate and haven’t been fully identified yet. This figure shows main cellular biological processes concerning atherosclerosis and CHD, which include AMPK-PI3K-Akt-eNOS, AKT/mTOR, p38 MAPK and ERK pathways to regulate inflammation, angiogenesis and other physiological activities. Irisin could also alleviate the damage of ROS directly. Solid lines represent promotion, and dashed lines represent suppression.

In recent years, thousands of papers are trying to find out irisin’s engages in multiple biological processes of our body (**Figure 1**). First of all, browning the white adipose tissue was recognized at the same time irisin was discovered in 2012 (1). However, as the discussion above, exercise has little effect on the stimulation of irisin’s browning function in human (15–20). Despite the disputes on stimulators like exercise, primary mouse adipocytes were observed browning effect when directly incubated with recombinant C-terminal FNDC5 peptide (25). Then in brown adipose tissue, the uncoupling protein 1 (UCP1) could release the storage of energy generated by oxidation in the form of heat and reduce the production of ATP simultaneously (26). These metabolic effects could be achieved by activating p38 MAPK and ERK pathways (27). The discovery of the PGC1α-FNDC5-irisin axis was the theoretical basis for the latter studies concerning energy metabolism mechanisms, which made irisin closely related to obesity, T2DM, and other metabolic syndromes (1, 28–30) (**Figure 2**). While the browning function is now facing challenges, similar questions would also be raised in related area such as fat storage, energy consumption and insulin sensitivity.

In addition, FNDC5 are associated with cognitive function of the brain through brain-derived neurotrophic factor (BDNF). BDNF is the main mediator of exercise effect on the hippocampus, which can promote the growth, differentiation and repair of neurons (31). Wrann showed a positive correlation between FNDC5 and BDNF in exercising mice (31). Increasing the brain level of FNDC5/irisin can enhance synaptic plasticity and memory in Alzheimer’s disease mouse models (32), and stimulate the STAT3 signal transduction pathway required for sensory neuron development (33). However, the studies didn’t explain the mechanism of serum irisin to cross the blood-brain barrier, and applied much higher concentration of irisin than physiological level, which later showed no obvious effects on mouse hippocampal neuronal cells (33). Thus, evidence is not enough to demonstrate the direct relationship of irisin and BDNF. Besides, in terms of bone metabolism, irisin were reported to activate proliferation and differentiation of osteoblast through p38 and ERK (34–36), and reduce the extent of osteoporosis and muscle atrophy (37, 38). But according to H Kim, the increase of trabecular bone volume and BMD were observed in female FNDC5-KO mice (39). So, we still couldn’t figure out whether irisin helps in bone formation or not. Also, the increase of irisin can be detected in a variety of tumors. In cell assays, irisin showed its potential to inhibit epithelial-mesenchymal transition and cancer cell invasion *via* PI3K/Akt pathway, enhance the activity of caspase-3 and caspase-7 to induce cancer cell apoptosis, and inhibit the activity of NF-κb to reduce inflammation (40, 41). Moreover, irisin produced by cardiomyocytes is much more than that of skeletal muscle cells (42). Thus, studies on treatment of irisin showed its effects on cardiac hypertrophy, mainly through mTOR activation to prompt protective autophagy (43–45). In the development of cardiovascular disease, irisin may participate in this process through various non-metabolic ways, which will be discussed in the latter part.

However, there has been a big shadow cast on the measurement of irisin. In 2015, Albrecht E showed that the antibodies used in commercial ELISA tests are unspecific with a huge variation in plasma concentrations (46), which could explain the huge difference between the early rough estimate by ELISA kits (1) and recent measurement by quantitative MS from the same group (21). Calibrated MS was regarded as a “gold standard” to detect irisin (47), but Albrecht further doubt the repeatability of LC/MS method for detection of extremely low plasma concentrations (48). Additionally, due to the extremely low amounts of irisin in samples (11, 21), it’s hard to purify it for gain-of-function experiments. Yet new approaches like recombinant irisin and FNDC5 knockout mice could provide more direct evidence to verify the functions of irisin. Most studies creating FNDC5 knockout mice found no obvious difference of multiple biological process under normal conditions (21, 43, 49, 50). But they would suffer a lot more in several abnormal conditions compared to wild type, such as fasting and high-fat diet (13, 43, 49, 51). Thus, we would put more focus on the studies with new approaches to draw a rather reliable picture of irisin.

### Coronary Heart Disease (CHD)

CHD is one of the most common cardiovascular diseases. The prevalence and fatality rate has been increasing consecutively year after year, seriously endangering people’s health (52). Due to coronary artery spasm or atherosclerosis, myocardial cells suffer from ischemia and hypoxia, leading to functional or structural pathological changes of heart. As early as irisin was first discovered, some scholars (53) suggested that there may be certain crosstalk between irisin and the cardiovascular system. As time passes by, a number of evidences accumulated to support this speculation. Irisin has participated in the development of CHD in a variety of ways, which is likely to lay a theoretical foundation for the clinical application of irisin as a biomarker or treatment.

## Irisin in Atherosclerosis

Atherosclerosis, a chronic inflammatory disease, is the most common pathogenesis of CHD and a variety of vascular diseases. The etiology has not been clear yet, usually considered to be closely associated with lifestyle and caused by multiple factors such as lipid metabolism disorder and endothelial injury (54). Irisin participates in the regulation of this process through direct or indirect ways (such as glucose and lipid metabolism).

### Controversies on Irisin’s Expression of Atherosclerosis

In many studies conducted in different sample sizes and different populations, the relationship between serum irisin levels and atherosclerosis is not consistent. Deng W et al. (55) conducted a cross-sectional study on the serum irisin concentration and coronary atherosclerosis index (CAI) of 350 coronary artery disease (CAD) patients and 214 healthy participants, finding that the serum irisin concentration was significantly different in the two groups of subjects. The lower the serum irisin concentration in CAD patients, the higher the CAI. Parallel to this result, in the study of cardiovascular complications, Khorasani (56) and Saadeldin (57) respectively confirmed that the serum irisin concentration was negatively correlated with the degree of coronary atherosclerosis in patients with type 2 diabetes. Besides, in some special populations [such as patients with dialysis (58) or Behcet’s Syndrome (59)], the detection of carotid artery intima-media thickness (IMT) was also found in line with this pattern. Irisin’s regulation on glucose and lipid metabolism is speculated to be the main contributor to this negative relationship, since hyperglycemia, insulin resistance and obesity are closely associated with atherosclerosis (55, 59, 60). Another possible explanation is the cross-talks between irisin and other molecules such as sclerostin or adipocytokines (57, 58).

However, some studies stood on the opposite side. As early as 2014, in a study of 192 European ancestry healthy people, it was found that irisin was positively correlated with the subjects’ carotid artery IMT (60). Moreover, a 25-year cohort study by Kwaniewska M (61) and a cross-sectional study of men with HIV infection (62) supported this result. In this regard, they explained that irisin may indirectly participate in the formation of atherosclerosis through fat or insulin (60, 62). And as the disease advances, the target cell’s sensitivity to irisin or insulin decreases, so that muscle/fat tissue “compensatively” releases more irisin to achieve the same effect as before. There is also an opinion, arguing that the difference in baseline between different studies leads to the opposite result: the various degrees of obesity of the study subjects may interfere with the results. As in non-obese subject, the serum irisin is mainly derived from muscle, while in the obese subject the proportion of fat source increases (63). Similar to the degrees of obesity, Moreno found that the high value of plasminogen activator inhibitor-1 (PAI-1) at baseline could also have a significant impact on the results, which could even count as an independent biomarker related to the thickness of the carotid artery IMT in patients (62). In a study based on a Japanese male population (64), the authors found that if cardiometabolic risk factors are taken into account, irisin had nothing to do with the prevalence of coronary artery calcification, but was related to progression. They believed that irisin may affect other unknown ways to cause this result except for traditional cardiometabolic risk factors such as fat and blood glucose. The inaccuracy measurement of irisin due to the questioned commercial ELISA may also count for the inconsistencies (46).

In short, the current studies lack consistency in the relationship between the level of irisin and the degree of atherosclerosis. And the correlation in multivariate analysis as well as the measurement is not sufficiently valid, which makes it difficult for irisin to be a predictive or clinical indicator of CHD.

### The Physiopathological Function of Irisin in Atherosclerosis

The cellular biological processes related to atherosclerosis mainly involves vascular endothelial damage and foam cell formation, which then leads to wall thickening, endothelial narrowing, and eventually fibrous cap rupture (54).

As mentioned above, irisin is surmised to participate in atherosclerosis by some indirect ways such as influencing insulin release. In recent years, a series of studies have found that irisin can also directly regulate endothelial function though suppressing the inflammation and oxidation and encouraging the proliferation of cells. On one hand, Lu J (65) proved that irisin can activate AMPK-PI3K-Akt-eNOS signaling pathway in diabetic mice, inhibit high glucose-induced apoptosis in human umbilical vein endothelial cells (HUVECs), and increase the expression of antioxidant enzymes to reduce inflammation and oxidative stress of endothelial cells. Furthermore, Han F et al. (66) conducted similar experiments on healthy obese mice fed by a high-cholesterol diet and found that the activation of AMPK-eNOS signaling pathway by irisin can be dependent or independent of adiponectin. On the other hand, Zhang Y et al. (67) elaborated in mice that irisin could reduce the vascular damage induced by oxLDL. In animal experiment, irisin can regulate the p38 MAPK/NF-κB signaling pathway by inhibiting the generation of reactive oxygen species (ROS) and the nuclear translocation of NF-κB. It can also inhibit PKC-β/NADPH oxidase, down-regulate inflammatory factors to reduce oxidative/nitrative stresses, thus alleviating vascular endothelial inflammation (67, 68). In addition, Zhang M et al. found that irisin can increase cell viability, cell migration and capillary formation in apolipoprotein E (ApoE) deficient mice. This may be achieved by the up-regulation of microRNA126-5p in the ERK signaling pathway so as to maintain the stability of endothelial cells and promote proliferation (69). In an *in vitro* experiment exploring diabetes complicated with vascular diseases, it reported that irisin can inhibit ROS-NLRP3 inflammasome signaling, thereby slowing down the process of diabetes-related endothelial inflammation and other impairment (70). Protective functions of irisin are also found to lessen endothelial damage at an early stage. Irisin could suppress the expression of TNFα-induced VCAM-1 in HUVECs (71). A recent survey in obese children found that irisin can inhibit the expression of hsCPR, ICAM-1 and E-selectin in endothelial cells, indicating that irisin may have an effect on cardiovascular disease before symptoms show up (72).

Besides endothelial cell, irisin could also act on macrophages to slow down the formation of plaques. By up-regulating Bcl-2, down-regulating the expression of Bax and caspase-3, irisin could prevent macrophages from turning into foam cells, and inhibit cell apoptosis (67). Zheng G et al. (73) further found lipid accumulation in macrophages cultured *in vitro* and oxLDL-related apoptosis were reduced under the interference of irisin, which may be related to the inhibition of PERK/eIF2α/CHOP and ATF6/CHOP endoplasmic reticulum stress signaling pathways. All of these demonstrated that irisin does engage in multiple mechanisms in atherosclerosis, either by acting on endotheliocyte or on macrophage. However, some of the experiments were conducted on mice, which may not be qualified enough to translate the protective effects to human. And it must be noted that the serum irisin level is much lower than the applied dose in experiment, thus challenging the physiological functions of irisin under normal circumstances.

### Therapeutic Effect of Irisin in Atherosclerosis

Various cell experiments have confirmed that irisin has a positive effect on vascular endothelial cells, while the therapeutic effect of irisin have shine a light on atherosclerosis in animal experiments. In apolipoprotein E-Null diabetic mice (69) or obese mice induced by high-cholesterol diet (66), the endothelium-dependent dilation function (EDV) was improved with the injection of irisin, while the endothelial cell apoptosis and the area of atherosclerotic plaque were pleasantly found to have a significant reduction. In the carotid artery partial ligation model (67, 69), systemic application of irisin can also inhibit the formation of the new carotid artery intima, for the reason that irisin may promote endothelial cell proliferation and inhibit monocyte recruitment and lipid deposition. These evidences show that irisin has potentiality to contribute to the therapy of atherosclerosis, while further researches are compulsory to provide sufficient clinical support.

## Irisin in CHD

CHD can be divided into two categories: stable angina pectoris (SAP) and acute coronary syndromes (CHD) according to its pathogenesis and clinical manifestations. Based on current studies, there is controversy over the role of irisin in the pathogenesis and repair of myocardial cells, and the specific mechanism is still unclear. But when it comes to treatment, most studies tend to support the protective effects of irisin on CHD.

### Fluctuation of Irisin During CHD

#### Limited Research of Irisin in SAP

SAP is the most common clinical type of CHD and is at the initial stage of CHD development. For many reasons, the insufficient blood supply of the coronary arteries leads to transient ischemia and hypoxia in the myocardium, often accompanied by cardiac dysfunction, but myocardial necrosis rarely occurs (74). There are not enough studies conducted on irisin in SAP, the quality of which are at different levels. In a small cross-sectional study, Efe et al. (75) found that the serum irisin level of stable coronary artery disease patients with a higher SYNTAX score (≥23) was significantly decreased compared to the group with low SYNTAX score or the healthy control group. They believed that serum irisin level can be used as an independent indicator to observe the severity of SAP, which is related to the degree of coronary stenosis in patients (76). Some previous researches (42, 77) also partly agreed with it. However, 24 hours after SAP patients having percutaneous coronary intervention (PCI), the serum irisin concentration was found to be lower than that of the control group (76). And the results of Park et al. (77) showed that compared with other groups, SAP patients with low preoperative serum irisin levels had a significantly higher proportion of no CHD events within 12 months after PCI. It should be noted that the measurement of irisin in those studies were all conducted by commercial ELISA, which reduced credibility of the potential connection between irisin and SAP, emphasizing the need for more clinical research.

#### Irisin’s Engagement in Acute Coronary Syndromes (ACS)

ACS happens after plaque rupture or erosion on the basis of coronary atherosclerosis, which leads to vascular embolism, a sharp decrease in oxygen supply to the myocardium, and then damage. The degree of risk and prognosis are related to the location, area and speed of the establishment of collateral circulation (78–80). Many studies believe that irisin is involved in the occurrence of ACS and can be used as a therapeutic drug.

Several studies reported that the concentration fluctuation of irisin had a common pattern in different stages of myocardial infarction (MI). Kuloglu et al. (81) used isoproterenol on rats to induce myocardial infarction. The serum irisin level of MI rats decreased significantly in the first 2 hours, and slowly increased afterwards, but never returned to the baseline within 24 hours during monitoring. In the meanwhile, the synthesis level in tissue like skeletal muscle, liver and kidney decreased within 1-4 hours after MI, and recovered after 6 hours. Further monitoring of myocardial infarction rats showed that the serum irisin level was highly positively correlated with QRS duration, amplitude and TAS, while highly negatively correlated with ST-elevation, QTc, CK-MB, troponin and MDA (82). Similar changing pattern can be observed in human that the concentration of irisin fluctuates during ACS, but the point of time was delayed (83, 84). Similarly, the serum irisin measured 6 hours after PCI also decreased from baseline (76). However, during coronary artery bypass surgery, the serum irisin concentration increased from induction to the removal of the cross-clamp, and then gradually decreased as the patient rewarmed (85).

According to this phenomenon, Kuloglu (81) believed that it had something to do with the uncoupling effect of irisin. and may have a protective effect on cardiomyocytes. In addition, excessive irisin will lead to increased mitochondrial respiration and fatty acid oxidation, which leads to increased oxygen consumption and ROS, and up-regulates the level of caspase 9 which enhances cell apoptosis (1). On the contrary, the appropriate amount of irisin can activate Opa1-induced mitochondrial autophagy, alleviate oxidative stress and maintain the vitality of cardiomyocytes after myocardial infarction (86). Irisin can also lead to bradycardia through effects on the nucleus ambiguous (87), which helps reduce the energy consumption of the heart.

However, different perspectives interpret the possible mechanisms behind the changes of irisin during ACS. One point is that the reduction of irisin is a passive performance rather than an active regulation. When cardiomyocytes were treated with TNFα or IL-1β, the expression of FNDC5 protein decreased significantly, indicating that inflammatory factors could inhibit the secretion of irisin (6). And MI can restrain the expression of PPAR-α and PPAR-γ (nuclear receptors), thereby preventing the interaction of PGC1-α with a variety of transcription factors, resulting in a decrease in the synthesis of FNDC5 (irisin precursor) (88). Another explanation suggested that the decrease in irisin level is the reason rather than the result of the decrease in coronary blood flow. Since previous animal experiments have proven that irisin can regulate endothelial function and induce vasodilation (89), low levels of irisin may lead to decreased vasodilatation then reduced blood flow, forming a vicious circle.

It should be noticed that the commercial ELISA kits used to detect irisin in those studies were lack of specificity (46). Besides the shadow of questioned measuring methods, the mechanism of this fluctuation still remains controversial. Sharp decrease of irisin may protect mitochondria and allow cardiomyocytes to save energy in such an ischemic environment, which seems to be a self-protection mechanism of our body. But it may be also a consequence of damage and even assist in the progression of ACS. While there is still no reliable method of irisin measurement, irisin is blocked to become a marker showing the process of ACS.

#### Invalid Prognostic Indicator

For the long-term stage after the occurrence of ACS, the level and function of circulating irisin are inconclusive. In terms of surgery, the level of irisin at 6 months after PCI was higher than the baseline (53). The relatively high level of irisin was positively correlated with the incidence of postoperative major adverse cardiovascular events (MACE), especially angina, the risk of which increased by almost 4 times (hazard ratio=3.96) (83). On the contrary, a meta-analysis involving 867 patients and 700 controls showed that patients with cardiovascular disease or atherosclerosis had significantly lower levels of irisin (90). In 2014, a cross-sectional study by Emanuele et al. (91) found that serum irisin in young subjects who had suffered from MI was significantly lower than healthy controls. However, in an age-related study (53), there was no significant difference in irisin levels among people in their sixties, regardless of whether they had had ACS. The measurement problem may be the main reason to rationale the difference (46). Moreover, The circulating level of irisin in the body is influenced by fat, exercise, diet and so many other factors, and the level of irisin tends to increase randomly due to aging (92). Meanwhile, systematic errors such as inconsistent experimental design and detection levels cannot be ruled out.

There are also studies concerning the distribution of irisin genotypes. The G allele of rs3480 and the A allele of rs726344 are significantly related to cardiometabolic risk factors. Besides, the two SNPs are associated with each other (93). Dyslipidemia is a major initiator of atherosclerosis, and ApoE is particularly important to maintain the normal metabolism of lipoprotein (94, 95). Fuku et al. reported that FNDC5 rs16835198 was associated with APOE ϵ2/ϵ4 allele (96), while direct evidence towards atherosclerosis needed to be further explored. Hence future investigations are needed to explore genetic risk factors for myocardial infarction.

Nevertheless, limited researches still couldn’t provide sufficient materials to make a conclusion, which suggests that a more critical experimental design and use of validated measurement are required to draw a rounded picture for prognosis of irisin.

### Irisin’s Potential in Treatment After Infarction

There is an interesting phenomenon in the distribution of irisin during myocardial infarction. Although serum irisin level decreases in the acute phase, the expression of irisin contrarily increases in connective tissues around the heart (81, 97), suggesting that irisin may participate in the follow-up process of MI such as restoration. As described above (67), we have mentioned that irisin can reduce the damage of vascular endothelial cells by inhibiting the production of ROS. Similar events may also occur in cardiomyocytes. Peng Q et al. (98) found that irisin can promote cell proliferation while reducing the production of HO-induced ROS and cell apoptosis in cardiomyocytes, which may be achieved by the miR-19b/AKT/mTOR signaling pathway. Zhao et al. (99) tried to find the connection between irisin and HDAC4 *in vitro*. HDAC4 is produced by H9c2 cardioblasts and is up-regulated in oxidative stress. Its overexpression can lead to mitochondrial dysfunction and even cell death (100). And irisin can induce HDAC4 degradation through ubiquitination modification to protect cardiomyocytes. Partially different from the results above (99), Moscoso et al. (97) only observed the protective effect of irisin under hypoxic conditions. They believed that under the condition of myocardial infarction, hypoxia and the compensatory adrenergic response would cause a lipotoxic environment and create conditions for apoptosis (101). Consequently, irisin could activate the Akt signaling pathway to resist apoptosis induced by lipotoxicity.

When the blood flow of myocardial tissue recanalized after ischemic injury, ischemia/reperfusion injury (I/R) often occurs. Irisin has shown its ability to reduce I/R injury *in vitro* and *in vivo* experiments. Mitochondrial dysfunction leads to the release of a large amount of ROS, and this oxidative stress response is one of the core steps of I/R. Irisin could inhibit the opening of mPTP(mitochondrial permeability transition pore), reduce mitochondrial swelling and increase the activity of superoxide dismutase 2 (SOD2) by upregulating mitochondrial ubiquitin ligase (MITOL/MARCH5), so as to protect mitochondria and reduce the oxidative stress response induced by mitochondria *in vitro (* 99, 102, 103).

Studies on the therapeutic effect of irisin are still ongoing. Irisin was used for treatment in mice with coronary artery obstruction caused by ligation. It indicated that irisin could activate the ERK signaling pathway and promote endothelial cell migration, thereby accelerating angiogenesis at the infarct border zone, reducing infarct size and fibrosis and improving ventricular diastolic function in animal model (104, 105), which may involve the AKT/mTOR/S6K1/Nrf2 pathway (106). The lack of those protective effects was also observed in FNDC5-knockout mice (13). Irisin can also assist the cardiac repair process. Deng et al. (107) used bone marrow mesenchymal stem cells (BM-MSCs) pretreated with FNDC5 to transplant into myocardial infarction mice. They reported that compared with non-pretreatment transplantation, irisin promoted the effect of BM-MSCs in inducing proliferation of myocardial cell and vascular, and significantly reduced myocardial remodeling and fibrosis as well as apoptosis signals. In addition, they also observed an increase in the survival rate of BM-MSCs and the secretion of exosomes. Chen et al. (100) believed that the repair effect may be related to HDAC4 degradation and p38 activation. This may show the possibility that irisin would improve the success rate of cell transplantation and provide a new direction for the treatment of MI.

However, the concentration used in experiments were much higher than physiological state, suggesting that irisin may not function in the restoration of heart itself, yet might be promising in medical treatment to reduce the damage of cardiomyocytes after infarction and help with cell transplantation of heart. New tools like recombinant irisin should be encouraged for higher credibility. More importantly, since the current researches concerning treatment are all conducted on cells or animals, whether irisin helps in human remains unknown.

## Discussion

Since irisin was discovered, people have conducted a lot of in-depth research on its relationship with metabolic diseases due to its most widely recognized uncoupling effect. In recent years, as the anti-inflammatory, anti-oxidant and anti-apoptotic effects of irisin have been exposed, more and more researchers are trying to explore the maximum value of this newfound molecule. In this review, studies have explored its potential in cardiovascular system (**Table 1**). Some *in vitro* and *in vivo* experiments have shown that irisin can regulate endothelial cell function and reduce the damage of atherosclerosis through various ways, which could be a sign of therapeutic use. During ACS, it was believed that an appropriate amount of irisin’s serum concentration can protect the myocardium from damage. The serum concentration drops first and then rises after MI, which may be related to the reduction of energy consumption and the protection of mitochondrial function. Nevertheless, irisin would enhance the repairing effect in the process of cell transplantation of heart, which showed its therapeutic potential in a new way.

**Table 1:** Functions of Irisin in CHD.

Effect	Experimental Subjects	Detail	Reference
Reducing endothelial inflammation and oxidative stress	HUVECs	Activating AMPK-PI3K-Akt-enos signaling pathway	(65, 66)
	HUVECs	Inhibiting p38 MAPK/NF-κB pathway	(67)
	HUVECs	Inhibiting PKC-β/NADPH oxidase	(68)
	HUVECs	Inhibiting ROS-NLRP3 inflammasome signaling	(70)
	HUVECs	Suppressing TNFα-induced VCAM-1	(71)
Promoting endothelial proliferation	HUVECs	Up-regulating microRNA126-5p	(69)
	HUVECs	Up-regulating ERK signaling pathway and suppressing high glucose-induced apoptosis	(109)
Reducing lipid accumulation in macrophages	RAW264.7 macrophages	Regulating Bcl-2, Bax and caspase-3	(67)
	HCAECs	Inhibiting the expression of hsCPR, ICAM-1 and E-selectin	(72)
	RAW264.7 macrophages	Inhibiting PERK/eif2α/CHOP and ATF6/CHOP ER pathways	(73)
Protecting cardiomyocytes	H9c2 cells, mouse ESCs	Inducing HDAC4 degradation to protect mitochondria	(97, 100)
	Primary cardiomyocytes	Activating mitochondrial autophagy	(86)
	H9c2 cells	Reducing the production of HO-induced ROS	(98)
	H9c2 cells	Inhibit the opening of mPTP	(99)
	H9c2 cells	Increasing the activity of SOD2	(102)
	H9c2 cells	anti-oxidation by the AKT/GSK3β/FYN/Nrf2 axis	(49)
	H9c2 cells	Activating AMPK pathway	(110)
	Sprague–Dawley rats	Causing bradycardia to reduce the energy consumption	(87)
Indicating the process of ACS	Rats or mice	Decreasing at acute phase after MI	(81, 82)
	Patients	Decreasing at acute phase after MI	(76, 83–85)
Inconsistencies in long-term monitoring of ACS	Patients	Increasing at 6 months after PCI	(53)
	Patients	Lower in MI patients	(91)
	Patients	Positively correlating to CHD events after PCI	(77)
	Patients	A positive predictor of MACE after surgery	(83)
	Patients	Irisin genotypes be considered as genetic risk factors of MI	(93)
Treating atherosclerosis	C57BL/6 mice, apoE-deficient mice	Improving EDV and reducing the area of atherosclerotic plaque	(66, 69)
	apoE-deficient mice	Inhibiting the new carotid artery intima’s formation	(67, 69)
Treating ACS	BM-MSCs	Enhancing the repairing effect in the process of cell transplantation of heart	(107)
	C57BL/6 mice	Accelerating the restoration of infarcted area	(104, 107)
	FNDC5 knockout mice	Accelerating the restoration of infarcted area	(105)

HUVEC, human coronary artery endothelial cell; ROS, reactive oxygen species; mPTP, mitochondrial permeability transition pore; SOD2, superoxide dismutase 2; MI, myocardial infarction; CHD, coronary heart disease; PCI, percutaneous coronary intervention; MACE, major adverse cardiovascular events; EDV, endothelium-dependent dilation function; apoE, apolipoprotein E; BM-MSC, bone marrow-derived mesenchymal stem cell.

Yet there has been a big limitation of irisin’s development in recent years. With the questioning of traditional measuring methods, lots of findings of irisin call for reconfirmation. The huge range of irisin level caused by inaccurate measurement led to the inconsistency of cross-sectional studies, making it hard for irisin to become a detection indicator (90). Besides, the restoration function of irisin in an infarcted heart is also facing challenges. The effective concentration applied to cells or mice was 10- to 8000-fold higher than physiological level in the gain-of-function experiments, which is not convincing enough to prove the physiological functions of irisin (108). In addition, due to the extremely low amount of irisin, purification is not easy to meet the demand.

With all those setbacks and a relative scarcity of preclinical and clinical data, we failed to draw a consistent conclusion of irisin’s function in CHD. Thus, future researches need to be careful of selecting measuring techniques as well as observation subjects in order to control the baseline. New methods like recombinant irisin and gene-knockout mice would be the development direction in function experiments. In addition, the studies have been too scattered, each team working own its own perspective. It would push forward a lot if a general framework would form. And the experiments at present still remained at the stage of small-scale animal experiments, indicating that higher levels need to be performed.

In brief, while irisin hasn’t been qualified enough yet as an indicator of CHD, it is quite promising in treatment based on current studies. Only with more considerations in experimental design and larger-scale studies will irisin show its true colors in the near future.

## Author Contributions

All authors listed have made a substantial, direct, and intellectual contribution to the work and approved it for publication.

## Funding

This work was supported by funding from the National Natural Science Foundation of China (Nos. 81770881 and 82070910) and Key R&D Plan of Hunan Province (2020SK2078).

## Conflict of Interest

The authors declare that the research was conducted in the absence of any commercial or financial relationships that could be construed as a potential conflict of interest.
